# Gamification, Exergames, and Digital Games in Older Adults Aged 75 and Over: Evidence on Well-Being, Quality of Life, and Social Engagement—A Scoping Review

**DOI:** 10.3390/healthcare14040470

**Published:** 2026-02-12

**Authors:** Dhurata Ivziku, Valentina Vanzi, Luca Guarente, Francesca Reato, Elisabetta Zuchi, Simona Ricci, Maria Ymelda Tolentino Diaz, Cesar Ivan Aviles Gonzales, Marzia Lommi

**Affiliations:** 1 Department of Healthcare Professions, Fondazione Policlinico Universitario Campus Bio-Medico, 00128 Rome, Italy; d.ivziku@policlinicocampus.it; 2 Department of Nursing, Faculty of Medical Science, AAB College, 10000 Pristina, Kosovo; 3 JBI Italy Evidence-Based Practice and Health Research Centre, 00136 Rome, Italy; 4 Center of Excellence for Nursing Scholarship (CECRI), Board of Nursing (OPI) of Rome, 00136 Rome, Italy; 5 Department of Biomedicine and Prevention, Tor Vergata University of Rome, 00133 Rome, Italy; 6 Department of Nursing Education, University of Insubria, 21100 Varese, Italy; francesca.reato@uninsubria.it; 7 Department of Health Professions, Azienda Sanitaria Locale (ASL) Roma 3, 00125 Rome, Italy; elisabetta.zuchi@aslroma3.it; 8 Department of Health Professions, Azienda Sanitaria Locale (ASL) Roma 2, 00159 Rome, Italy; 9 Department of Medicine and Surgery, Kore University of Enna, 94100 Enna, Italy

**Keywords:** very old, oldest-old, exergames, serious games, gamification, virtual reality, well-being, quality of life, scoping review, age stratification

## Abstract

**Highlights:**

**What are the main findings?**

- In adults aged ≥75, evidence on game-based digital interventions is concentrated in supervised exergames, evaluated mainly through physical-function measures, with QoL and social outcomes reported less consistently.
- A persistent gap is limited age-disaggregated reporting, as many mixed-sample studies do not provide extractable results for the ≥75 subgroup.

**What are the implications of the main findings?**

- Studies should routinely report age-disaggregated outcomes (≥75 vs. <75) and use multidimensional, standardized outcome sets that include QoL and social well-being.
- Implementation research should test safe, acceptable delivery for ≥75 populations and compare supervised institutional models with scalable home/community formats.

**Abstract:**

**Background/Objectives**: Population ageing is accelerating, and adults aged ≥75 years (the “very old”) have distinct functional, cognitive, and social needs. Game-based digital interventions—including exergames, serious/digital games, and gamification—may promote multidimensional well-being; however, findings are often reported for broad “older adult” samples without age-disaggregated results. This scoping review mapped the use and effects of game-based digital interventions in adults aged ≥75 years and assessed how frequently studies reported age-specific outcomes. **Methods**: The review was conducted in accordance with JBI guidance. PubMed, CINAHL, PsycINFO, Scopus, and Web of Science were searched. Records were screened in Rayyan by two reviewers. Data were extracted using a standardized charting form, and outcomes were classified into predefined outcome domains and implementation-related categories. **Results**: Nineteen studies were included, predominantly European and largely conducted in supervised institutional settings. Exergames were the most common intervention type. Physical outcomes were reported most frequently, whereas cognitive, emotional, social, and quality-of-life outcomes were assessed less consistently. **Conclusions**: In adults aged ≥75 years, evidence on game-based digital interventions is mainly based on supervised exergame programmes and emphasizes physical-function outcomes, while age-disaggregated reporting and person-centred outcomes remain limited. Future research should routinely report results specific to participants aged ≥75 and broaden outcome assessment to better inform intervention design for the very old.

## 1. Introduction

Population ageing represents one of the most profound demographic shifts of the 21st century. The World Health Organization (WHO) estimated that in 2019 around one billion people worldwide were aged 60 years or older, with projections rising to 2.1 billion by 2050 [1,2]. In parallel with increasing life expectancy, however, the burden of chronic conditions and frailty also grows, placing substantial pressure on health and social care systems [3]. Ageing is a multifaceted process influenced by biological, psychological, social, and environmental factors. Consequently, later-life well-being should not be framed merely as the absence of disease; rather, it reflects a dynamic equilibrium among cognitive health, functional independence, and psychological stability. When one dimension declines, ripple effects often emerge across the others, ultimately shaping overall quality of life [4].

From a physical and functional standpoint, muscle strength, mobility, and balance are central to safety and independent living. Reductions in these capacities are linked to fear of falling, greater reliance on assistance for daily activities, and diminished autonomy [5,6]. Cognition encompasses memory, attention, executive functioning, and processing speed, typically measured through neuropsychological assessment. Age-related cognitive changes may evolve into mild cognitive impairment—an intermediate stage between typical ageing and dementia—highlighting the importance of targeted cognitive stimulation and support [7]. Psychological well-being is equally important: mood, self-esteem, resilience, and self-efficacy contribute meaningfully to overall well-being, and stronger confidence in one’s capabilities has been associated with better quality of life and more effective adaptation to age-related transitions [4]. Social well-being—capturing relationship quality and community participation—can influence health outcomes to a degree comparable to clinical conditions. Social isolation, which is distinct from the subjective experience of loneliness, is widely recognized as an independent risk factor for adverse health outcomes, with effects comparable in magnitude to those of chronic diseases [8,9]. The environment further shapes autonomy and well-being: accessibility, safety, and the availability of technological supports may function as either facilitators or barriers [9]. Within this broader perspective, social determinants of health—such as education, income, housing, and access to services—play a decisive role in shaping trajectories of ageing [10].

These multidimensional elements align with the WHO active ageing paradigm, which advocates integrated strategies that sustain health and participation across the life course. Within this framework, approaches such as gamification, exergames, and digital games have increasingly been explored as tools capable of stimulating physical, cognitive, and social domains simultaneously [11]. Over recent years, digital technologies have been positioned in public health as potentially valuable resources to promote active ageing. Evidence suggests that well-designed interventions may contribute to improvements across several domains, including reduced frailty and slower functional decline in older populations [7,11,12]. Among older adults with mild cognitive or physical impairment, technology-based solutions implemented in clinical and community settings have been described as supporting executive functions, mobility, balance, memory, and attention. Notably, interactive formats may strengthen motivation and continuity of practice, thereby improving adherence to stimulation or rehabilitation programmes [4,6,7]. Rather than replacing traditional approaches, these tools may provide engaging and stimulating scenarios, with potential positive effects on well-being and quality of life [5,7].

Malnutrition is common in late life and may compromise functional capacity and exercise tolerance, potentially shaping engagement and adherence in physically demanding exergaming interventions. Nutritional risk in older inpatients has been associated with measurable blood parameters, including hematological/immune markers and oxidative-stress/adipokine profiles. This clinical context is relevant when interpreting feasibility and responsiveness in adults aged ≥75 years [13,14].

Within the broad umbrella of health-oriented “digital games”, we use “digital games” as an overarching term for interactive game-based software delivered via consoles, computers, tablets/smartphones, or immersive platforms, including both commercial games adapted for health purposes and serious games designed primarily to achieve educational, rehabilitative, or therapeutic goals. Among older adults, serious games are commonly used for cognitive stimulation (e.g., memory, attention, problem-solving) and have been associated with improvements in cognitive outcomes and selected mood-related measures in populations with cognitive impairment, although effects vary across studies and interventions [7,15]. Exergames (active video games) represent a subset of digital games that integrate physical exercise with gameplay through motion sensors or movement-tracking technologies (e.g., Nintendo Wii™/Wii Fit™, Kinect™, and VR-based exergames), aiming to support mobility while enhancing enjoyment and engagement. Evidence syntheses in older adults indicate that exergaming is generally feasible and usable and may benefit physical activity, balance, and functional outcomes, with positive user experiences reported as potential facilitators of adherence [6,12,16]. Finally, gamification refers to the application of game design elements (e.g., points, levels, badges, challenges, and real-time feedback) to non-game health activities (e.g., home exercise or self-management tasks) to increase motivation and adherence [11]. Although these approaches can overlap, they differ in purpose, interactivity, and expected effects across cognitive, motor, and motivational domains; clear conceptual boundaries are therefore essential to align intervention choice with individual profiles and clinical or educational objectives.

Alongside these approaches, virtual reality (VR) is increasingly used in the field of interventions for older adults; however, in this scoping review VR is not conceptualized as a standalone intervention category, but rather as an immersive technology platform/delivery modality that can be used to implement game-based content (e.g., VR-based exergames or VR serious games). Accordingly, we considered VR-based interventions eligible only when they incorporated explicit game elements (e.g., rules/challenges, performance feedback, progression/rewards) and active user interaction consistent with digital game–based approaches, whereas VR applications delivered solely as non-game therapeutic modalities (e.g., passive exposure/relaxation/distraction without a game structure) fall outside the scope of the present review. This positioning supports a coherent mapping of game-oriented interventions while acknowledging VR as a rapidly expanding modality that may simultaneously target cognitive and motor functions and support engagement in later life [5,8].

Despite growing interest, the field still faces notable methodological and content-related challenges. Many studies employ small samples and short follow-up durations, limiting conclusions regarding long-term sustainability. Outcome standardization is often insufficient: while neuropsychological measures and motor indicators are frequently used, person-centred outcomes—such as quality of life, perceived well-being, and social participation—are assessed less consistently [5,7,8]. Another recurring limitation is the lack of systematic stratification by age, frailty, comorbidity burden, and digital literacy, which reduces comparability across studies and weakens transferability to real-world contexts [6,11,17].

This work focuses specifically on the “very old” (≥75 years), a threshold commonly used to capture a later-life subgroup with substantially higher probability of functional limitations and need for assistance compared with adults aged 65–74, which can critically influence feasibility, safety, and responsiveness to digital/game-based interventions [3].

Older populations are inherently heterogeneous, and distinguishing the young-old (65–74 years) from the very old (≥75 years) reflects meaningful differences in functional reserve, frailty, clinical profiles, and familiarity with technology. When studies pool broad “older adult” samples (often defined as ≥65 years) and report aggregated outcomes without age-disaggregated analyses, findings can be difficult to interpret for the very old and may obscure subgroup-specific patterns in feasibility, acceptability, and outcomes. In practice, this may lead to interventions that are less effective—or less transferable—for those at highest risk of functional decline. A sharper focus on evidence specific to adults aged ≥75 is therefore essential to support more targeted, equitable, and personalized digital interventions.

Against this background, a scoping review is warranted to systematically map the available evidence on the use and effects of gamification, exergames, and digital games in older adults, with particular attention to the very old (≥75 years). Such mapping can clarify which intervention types have been investigated, which outcomes have been prioritized, and the extent to which studies stratify samples or findings by age (≥75 vs. <75 years). Ultimately, this synthesis can provide an integrated overview of the state of the art and guide the development of future studies with more robust, consistent, and practice-relevant protocols aligned with active ageing and public health priorities.

## 2. Materials and Methods

### 2.1. Design

This scoping review was conducted in accordance with the Joanna Briggs Institute (JBI) methodological guidance for scoping reviews [18] and is reported following the PRISMA-ScR checklist [19]. The protocol was developed a priori and registered in the OSF platform (DOI https://doi.org/10.17605/OSF.IO/3UK28) [20].

### 2.2. Research Questions

The review was guided by the following primary question: “What evidence is available on the use of gamification, exergames, and digital games to support health and well-being in older adults aged ≥75 years, and what intervention characteristics and outcome domains are reported?”.

The secondary questions were:

*1.*  How are gamified and digital interventions applied among adults aged ≥75 years?
*2.*  Which outcomes are reported for this age group (e.g., quality of life; physical, cognitive, emotional, or social well-being)?
*3.*  To what extent do studies explicitly stratify samples or results by age, providing findings for those aged ≥75 years?

The research questions and eligibility criteria were informed by the JBI Population–Concept–Context (PCC) framework. The Population comprised adults aged ≥65 years, with subgroup reporting or extractable data for participants aged ≥75 years. The Concept included gamification, serious games, exergames, or other digital games targeting outcomes related to quality of life, socialization, and well-being. The Context encompassed community, home, residential, and healthcare settings, with no geographical restrictions (Supplementary Table S1).

### 2.3. Eligibility Criteria

Eligibility criteria were defined a priori to map evidence on game-based digital interventions relevant to the oldest-old (≥75 years). Primary empirical studies (quantitative, qualitative, or mixed-methods) were eligible if they evaluated the use and/or effects of gamification, exergames, serious games, or other digital games intended to improve quality of life, well-being, and/or social interaction. Studies were eligible when they involved adults aged ≥65 years and either: (i) reported subgroup analyses for participants aged ≥75 years, or (ii) provided extractable data enabling separate identification of results for the ≥75 subgroup. Both community-dwelling and institutionalized populations were eligible, including participants recruited in clinical, rehabilitative, or community settings.

Interventions were required to include a digital, gamified, or game-based component (e.g., videogames, exergames, serious games, or gamification features delivered through digital platforms).

When VR was involved, studies were eligible only if VR was used to deliver a game-based or gamified intervention (e.g., VR exergames/VR serious games); studies using VR solely as a non-game therapeutic modality were excluded.

No restrictions were applied by country, publication date, or language; setting was not used as a restriction.

Studies were excluded if the entire sample was <65 years or if results specific to adults aged ≥75 years were neither reported nor extractable. Interventions without a digital/game-based component (e.g., purely analogue activities, non-gamified educational programmes, or physical training without game elements) were also excluded. Non-empirical publications (e.g., protocols, editorials, opinion pieces, conference abstracts, and theoretical papers) were excluded if they did not provide primary data consistent with the PCC criteria, particularly regarding digital/game-based interventions and outcomes related to quality of life, well-being, and/or socialization.

This approach was intended to maximize sensitivity by initially considering studies including adults aged ≥65 years, while maintaining an analytic focus on the ≥75 subgroup.

### 2.4. Search Strategies and Study Selection

A comprehensive search strategy was developed to maximize coverage while ensuring transparency and reproducibility. The strategy was designed and refined in consultation with a health sciences librarian, in line with JBI guidance.

The most recent electronic database searches were conducted in November 2025 in PubMed, CINAHL, PsycINFO, Scopus, and Web of Science. Search terms combined controlled vocabulary (where applicable) and free-text keywords across three domains: (i) older adults (≥65 years), (ii) gamification/digital games/exergames/serious games, and (iii) outcomes related to quality of life, well-being, and/or social participation. Full search strings and database-specific queries are reported in Supplementary Table S2.

Because many studies define “older adults” as individuals aged ≥65 years, the search was not restricted to ≥75 years at the retrieval stage. Instead, the age-specific focus (≥75 years) was applied during screening and full-text eligibility assessment.

Records were managed and screened using Rayyan [21], enabling independent title/abstract screening and full-text assessment by two reviewers. Disagreements were resolved through discussion, and a third reviewer adjudicated when needed. Reasons for full-text exclusions are reported in Supplementary Table S3.

### 2.5. Data Extraction

Data were charted using a standardized extraction form developed for this review to capture study characteristics and age-specific reporting for adults aged ≥75 years. Extracted variables included: author(s), year, country/continent and technology/intervention type (e.g., exergame, serious game, digital game, gamification, including virtual reality–based approaches where relevant); study design and aims; participant characteristics (sample size, sex distribution when reported, age range, mean/median age, and the proportion/number of participants aged ≥75 years when available); and data collection methods and instruments (e.g., performance-based measures, questionnaires, interviews, and device/app tracking).

To improve reproducibility, we applied explicit operational rules to classify interventions when charting the technology/intervention type. Studies were coded as exergames when gameplay required and monitored body movements/physical exercise (including VR exergames); as serious games when a complete digital game was purpose-built for a health, educational, or rehabilitative aim (including VR serious games); as digital games when commercial or non-purpose-built games were used/adapted for health-related outcomes; and as gamification when game design elements were applied to a non-game activity without a full game environment. For hybrid interventions, the category reflected the dominant game component/primary mechanism described by the authors; disagreements were resolved by consensus.

Outcomes were extracted and coded into predefined domains aligned with the review questions: quality of life and physical, cognitive/mental, emotional/affective, and social well-being. In addition, given the prominence of acceptability-related outcomes across the included studies, indicators of engagement/adherence, feasibility/safety (e.g., dropouts, adverse events), and usability/experience (e.g., SUS/SSQ or qualitative user feedback) were systematically charted when reported.

The extraction form was piloted independently by two reviewers on a subset of included studies and subsequently refined iteratively to improve clarity and consistency. Any discrepancies were addressed through discussion and consensus.

### 2.6. Data Synthesis and Presentation

Consistent with the objectives of a scoping review, no formal critical appraisal of methodological quality or risk of bias was undertaken. The aim was not to judge methodological rigour, but to map the extent, nature, and characteristics of the available evidence on gamified and digital interventions for older adults, with a specific focus on those aged ≥75 years.

Extracted data were analyzed and presented descriptively, drawing on both quantitative and qualitative approaches. First, a world map was used to illustrate the geographical distribution of the included studies. Second, study characteristics were summarized using counts and proportions (e.g., distribution by country/continent, study design, and intervention technology) and presented in tables, with an accompanying frequency heatmap for visual overview. Third, outcome findings were narratively synthesized and structured by outcome domain (quality of life; physical; cognitive/mental; emotional/affective; social well-being) and by intervention type (exergames, serious games, digital games, and gamification-based approaches). Indicators of engagement/adherence and feasibility/usability were summarized alongside outcome results to contextualize implementation and acceptability in the ≥75 population. A visual mapping was also developed to support interpretation of patterns across studies.

Taken together, these descriptive and visual syntheses highlight areas of convergence and divergence across intervention categories and outcome domains and facilitate identification of evidence gaps and underexplored outcomes among adults aged ≥75 years.

## 3. Results

### 3.1. Selection Process

**Figure 1:**
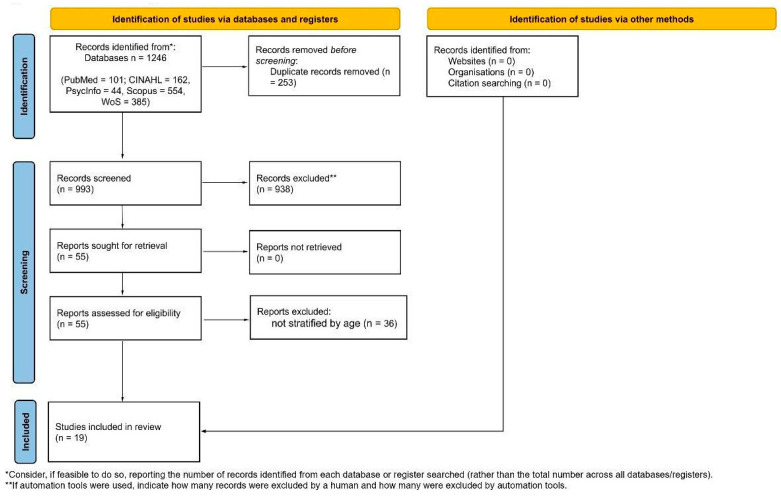
PRISMA-ScR flow diagram [19].

A total of 1246 records were retrieved from PubMed, CINAHL, PsycINFO, Scopus, and Web of Science databases (see Supplementary Table S3). After removing 253 duplicates, 993 records underwent title and abstract screening, and 938 were excluded as they did not meet the inclusion criteria. Fifty-five full-text articles were assessed for eligibility. Of these, 36 were excluded because, although they included older adults, results were not stratified by age, and findings specific to participants aged ≥75 years were neither reported nor extractable. Ultimately, 19 studies were included in the final scoping review. The selection process is summarized in the PRISMA-ScR flow diagram (see Figure 1).

### 3.2. Characteristics of the Included Studies

**Figure 2:**
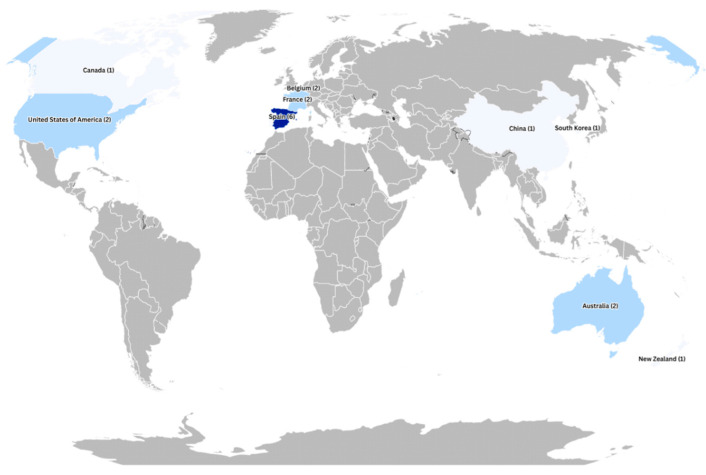
Geographical distribution of studies included in the review.

Nineteen studies included participants aged ≥75 years. Overall, the geographical distribution of the evidence was predominantly European. Twelve studies examined digital technologies designed to support physical functioning, cognitive stimulation, and psychosocial well-being (seven conducted in Spain, two in Belgium, two in France, and one in Portugal). Four North American studies (three from the United States and one from Canada) investigated digital interventions implemented in clinical and residential settings, with a particular emphasis on user experience and engagement among older adults. Three studies from Oceania (two in Australia and one in New Zealand) evaluated the effectiveness of exergames and digital games in improving motor functions, enhancing motivation for physical activity, and fostering social participation. In Asia, two studies (one in China and one in South Korea) focused on home-based programmes or interventions for older adults with dementia, aiming to improve physical, cognitive, and emotional outcomes and to reduce depressive symptoms (see Figure 2).

Regarding study design, most included articles (12/19) adopted interventional/experimental approaches. Ten were randomized controlled trials (RCTs), while two were non-randomized interventional studies (1 quasi-experimental study and 1 controlled, open-label, non-randomized clinical trial). The remaining studies included three pilot/observational studies, two mixed-methods pilot studies, one qualitative study, and one cross-sectional study, largely aimed at evaluating the feasibility, usability, and user experience of digital technologies.

**Figure 3:**
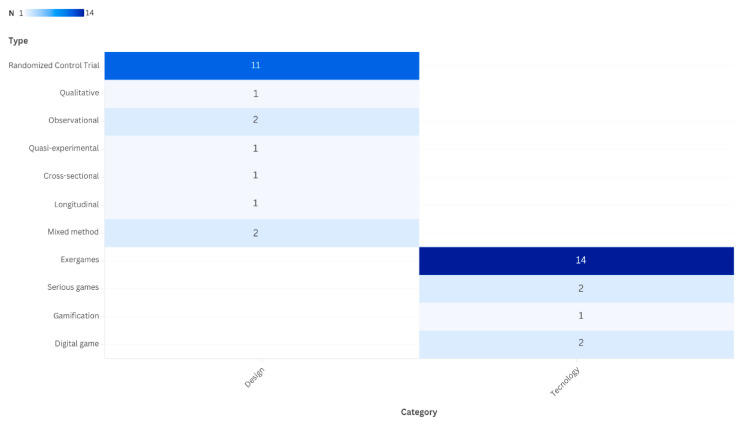
Distribution of the number of studies by study design and technology.

With respect to the technologies employed, exergame platforms were most common (*n* = 14), combining physical activity with game-based digital elements to promote motor exercise and, in some cases, cognitive training. Two studies evaluated serious games, primarily targeting cognitive or music-based stimulation, and two tested digital games, including immersive virtual reality applications. Finally, one study used a gamification approach to assess the effects of game mechanics on functional capacity in hospitalized older adults (see Figure 3). A detailed overview of the included studies is provided in Table 1.

### 3.3. Synthetic Summary of Instruments and Direction of Findings

In the studies reviewed, various instruments were used to assess quality of life and social well-being outcomes (see Table 1). For quality of life, instruments such as the SF-36, SF-12, EQ-5D-3L, EUROHIS-QOL, and QoL-AD were commonly employed, with most studies reporting improvements in the mental component of quality of life, particularly in studies involving exergames and cognitive stimulation. However, the physical component of quality of life showed mixed results, with some studies indicating improvements while others did not find significant changes. Social well-being was assessed using tools like the Lubben Social Network Scale (LSNS) and the social functioning subscales of the SF-36, and findings generally indicated increased social interaction, bonding with peers, and greater participation in social activities, especially in group-based interventions. However, deeper or more sustained social connections were less frequently observed. Emotional and affective well-being, measured through instruments like the Geriatric Depression Scale (GDS) and emotional well-being subscales of the SF-36, typically showed a reduction in depressive symptoms, especially in studies with exergames and cognitive training. Emotional well-being was generally found to improve, though the results varied in statistical significance.

### 3.4. Study Settings and Populations

Across the 19 included studies, participants were most commonly recruited from institutional and supervised settings—such as assisted-living facilities, residential care homes, and long-term care/nursing homes—with many interventions delivered in group formats under staff supervision. Fewer studies involved community-based or home-based participation, including one home-based exergame programme targeting community-dwelling older adults. One interventional study took place in an acute hospital setting and examined a gamification approach implemented during hospitalization. Participant characteristics generally reflected the oldest-old population.

Mean ages were often in the mid-80s, and several studies included participants in their late 80s or early 90s, including nonagenarians. Samples were frequently female-predominant, consistent with the demographic composition of older adults in residential and long-term care environments. Clinical characteristics varied across studies. Some investigations specifically targeted older adults with cognitive impairment or dementia, particularly within long-term care or memory-care contexts, whereas other enrolled older adults without a defined clinical condition or included mixed samples (e.g., with and without cognitive impairment). Overall, the evidence spanned a wide range of functional and clinical characteristics—from frailty and mobility limitations to dementia-related cognitive and behavioural symptoms—highlighting the heterogeneity of adults aged ≥75 years receiving digital game-based interventions.

**Table 1:** Characteristic of studies included (n. 19).

N	Author/Year, Country/ Continent, Technology	Design Aim (s)	Sample	Data Collection	Results
1	Chao et al., 2015 [22] USA (North America) Exergame (SAHA programme + Wii Fit)	Quasi-experimental study To examine the effects of the SAHA programme, which integrates self-efficacy theory into Wii Fit exergames, on physical function, fear of falling, depression, and quality of life among older adults living in residential care facilities.	32 participants divided into 2 groups: **EG** (16; 5 males, 11 females; 86.63 ± 4.18 years) **CG** (3 males e 13 females; 83.75 ± 8.04 years)	The following variables were measured at baseline (T0) and after 5 weeks (T1): – Physical function: BBS-14, TUG, 6MWT – Depression: GDS-15 – Quality of life: SF-8 – Self-efficacy: FES, SEE	**Physical function:** BBS ↑ from 40.53 ± 6.59 to 43.93 ± 6.34 → *p* < 0.01 TUG ↓ from 18.52 ± 5.60 to 15.27 ± 4.68 → *p* < 0.01 6MWT: no significant difference (*p* = 0.12) **Depression:** GDS-15 ↓ from 2.67 ± 2.44 to 2.00 ± 2.04 → *p* = 0.02 **Quality of life:** SF-8 PCS: no significant difference (*p* = 0.60) SF-8 MCS: upward trend ↑ (50.75 → 52.98) → between group *p* = 0.04 **Self-efficacy:** No significant effects **Physical well-being:** Improved balance and mobility **Emotional well-being:** Reduced depressive symptoms, increased confidence and enjoyment **Mental well-being:** Trend toward improvement in the mental component of quality of life **Social well-being:** Indirect effects observed (greater interaction, peer support, mutual encouragement) **Quality of life:** Improvement in the mental component (MCS) **Engagement:** Hight → Residents were motivated to continue; administrators were asked to keep the programme running
2	Chao et al., 2016 [23] USA (North America) Exergame (Nintendo Wii/Wii Fit)	Qualitative study To explore the facilitators and barriers perceived by older adults in assisted living regarding the use of exergames, with particular attention to cognitive, physical, and psychosocial effects, applying self-efficacy theory	15 participants (11 females and 11 males; 87.1 ± 3.9 years; range 78–92 years)	Semi-structured interviews	**Facilitators:** **Health and mobility** (staying active, weight control, increased energy) **Greater alertness and attention** (a “sharper” mind) **Enhanced psychological well-being** (hope, sense of achievement, enjoyment) **Social interaction** (fun, bonding with peers and family members—including grandchildren—reducing the generational gap) **Structured programme** (commitment, goals, supervision) **Barriers:** **Physical and cognitive limitations** (sensory impairments, arthritis, post-stroke effects, coordination difficulties) **Unpleasant experiences** (fear of falling, fatigue, pain, frustration with negative game feedback) **Physical well-being:** Feeling stronger, fitter, with greater energy and improved mobility **Cognitive/mental well-being:** Exercise keeps the mind active and increases attentiveness **Emotional well-being:** Hope, joy, entertainment, sense of accomplishment **Social well-being:** Strengthened bonds with peers, increased interactions, intergenerational connections **Quality of life:** Perceived as improved due to new, stimulating, and shared activities **Engagement:** High, with 80% wishing to continue using Wii exergames and recommending them to family and friends
3	Yasini & Marchand 2016 [24] France (Europe) Serious game (mobile cognitive-training app)	Observational pilot study To evaluate the adoption and perceived effectiveness of a mobile health app for cognitive stimulation in older adults, in terms of usage, game performance, and perceived well-being	15 participants (9 females and 6 males; range 79–88 years)	The following parameters were assessed at 1 month (T0) and 6 months (T1): -Automatic app tracking: gameplay time, number of sessions, difficulty level, success rate -Self-reported well-being (Likert scale 1–6, from “not at all satisfied” to “very satisfied”), completed at the end of each session.	**Cognitive/mental well-being:** Indirect improvement through better performance and success in the games **Emotional–affective well-being:** Perceived increase in enjoyment and satisfaction associated with continued use **Engagement:** High; no loss of interest over 6 months, increasing use, and good acceptability
4	Mugueta-Aguinaga & Garcia-Zapirain, 2017 [25] Spain (Europe) Exergame (FRED)	Randomized clinical trial (pilot study) To analyze the use of the FRED game, designed to support frail older adults	39 participants divided into 2 groups: **EG** (20; 60% females and 40% males; 85.47 ± 6.46 years) **CG** (19; 60% females and 40% males; 83.11 ± 9.01 years)	The following variables were measured at T0 and after 3 weeks (T1): -Physical function: SPPB -Acceptability and motivation: ad hoc questionnaires	**Physical function:** -The EG showed a significant improvement (*p* < 0.001), while the CG worsened -60% of the EG (12/20) achieved a score ≥ 10 (no longer at risk of frailty) -85% of the EG demonstrated improvement **Engagement/Adherence:** **-**100% compliance with the sessions **-**After the first 2 days, 100% positive responses regarding acceptability and motivation **-Physical well-being:** Significant improvement in balance and functional mobility (SPPB) **-Emotional well-being:** Increased motivation and high levels of acceptability
5	Taylor et al., 2018 [26] New Zealand (Oceania) Exergame (group-based programme; platform NR)	Randomized clinical trial To examine whether a group-based exergame programme improves mobility in older adults living in long-term care facilities, with and without cognitive impairment	65 participants divided into 2 groups: **EG** (26; 20 females and 6 males; mean age 86.7 years; range 78–90) **CG** (32; 23 females and 9 males; mean age 85.8 years)	The following variables were measured at T0 and after 8 weeks (T1): **-**Global mobility: DEMMI -Functional mobility and fall risk: TUG -Physical activity in daily life: wearable electronic devices (Dynaport Move Monitor)	**Mobility:** improvement in the exergame group (+6.3 points, trend *p* = 0.06, not significant) **Cognition:** did not influence outcomes **TUG:** no significant difference **Physical activity:** no difference between ES and CG. **Adherence:** average frequency 55% (8.8 ± 5.2 sessions out of 16); 27% attended ≥14 sessions. **Well-being and socialization:** participants reported enjoyment, motivation to stay active, a positive sense of obligation to attend sessions, and interaction with the instructor and peers. No adverse events.
6	Coelho et al., 2020 [27] Portugal (Europe) Digital game (360° VR reminiscence experience)	Mixed methods pilot study Assessing the effects of using virtual reality headsets with 360° vision to stimulate memories in people with dementia	9 participants (6 females, 3 males; 85.6 ± 7.4 years)	Tests were used to investigate the following variables: -Quality of life: EUROHIS-QOL-8 -Behavioural symptoms: NPI -Emotional reactions: observational scales -Malingerer syndrome: SSQ -Caregiver opinions: semi-structured interviews	**Engagement:** 73.5% very interested in exploring the environment 57.7% communicated spontaneously 71.1% shared personal memories 56.2% recalled positive/happy memories Experience rated as ‘pleasant’ in 83% of sessions **Behavioural/neuropsychiatric symptoms**: No significant increase. Mild anxiety, agitation, irritability in a few cases (transient, not severe) **Quality of life:** No significant change **Simulator sickness:** No severe cases. Only mild/intermittent symptoms (e.g., visual fatigue, feeling of fullness in the head, blurred vision) **Emotional well-being:** pleasure, positive emotions, temporary reduction in apathy **Mental well-being:** stimulation of autobiographical memory, recall of memories **Social well-being:** increased communication with carers and researchers, greater curiosity
7	García Bravo et al., 2020 [28] Spain (Europe) Exergame (Wii Fit Plus)	Prospective longitudinal study Assessing the impact of Wii Fit Plus training on dynamic balance and quality of life	12 participants (80.75 ± 3.66 years)	The following variables were measured at T0 and after 4 weeks (T1): Dynamic balance/postural control: Smart Equitest System (LOS test: RT, MVL, EPE, MXE, DCL) Quality of life: SF-36	**Dynamic balance:** Improved RT in backward and oblique movements (*p* = 0.012, 0.050, 0.021) MVL ↑ in forward and oblique movements (*p* between 0.012 and 0.028) EPE ↑ in backward and oblique movements (*p* = 0.012, 0.043, 0.050) MXE ↑ in multiple directions (*p* between 0.012 and 0.042) DCL ↑ in backward movement (*p* = 0.042) **Quality of life:** Improvements in Vitality (*p* = 0.046) and Role Emotional (*p* = 0.038) Positive trend in Social Function and Mental Health, but not significant **Physical well-being:** improved dynamic balance and postural control **Emotional well-being: increased vitality, reduced emotional limitations** **Mental well-being**: positive trend (MH) but not significant **Social well-being:** perceived improvement but not significant **Engagement:** high → programme well tolerated, motivating, safe with physiotherapist supervision
8	McCord et al., 2020 [29] Australia (Oceania) Digital game (commercial action video games)	Randomized clinical trial (pilot study) To examine whether short sessions of action video games improve visual attention, task switching, working memory and quality of life in the oldest-old (≥80 years) residing in long-term care facilities	24 participants (19 females and 5 males; 89.17 ± 4.01) divided into EG (12) and CG (12)	The following variables were measured at T0 and after 3 weeks (T1): -Attention: TMT-A/B -Working memory: DSLNS, WMS-III -Quality of life: OPQoL-Brief	**Visual attention:** significant improvement in the intervention group, maintained and increased at follow-up (*p* = 0.015). **Task switching:** significant improvement, maintained at 1 month (*p* = 0.012). **Working memory:** **Digit Span forward** ↑ significant (*p* = 0.030) **Digit Span backward** ↑ at post-test (*p* = 0.028) **Letter-Number Sequencing** ↑ at post-test (*p* = 0.004) **Quality of life:** no significant differences between groups or over time. **Mental well-being:** improved executive processes (attention and cognitive flexibility). **Engagement:** high, low attrition, intervention well tolerated and enjoyable for participants.
9	Jahouh et al., 2021 [30] Spain (Europe) Exergame (Wii Fit)	Randomized clinical trial To determine the impact of using Wii Fit on the performance of activities of daily living and the cognitive and psychological status of institutionalized elderly people	80 participants divided into 2 groups: **EG** (40; 22 females and 18 males; 85.05 ± 8.63 years) **CG** (40; 23 females and 17 males; 83.25 ± 8.78 years)	Tests and questionnaires were used to investigate the following variables: -Cognitive functions: MCE -Depression: Global Deterioration Scale -Activities of daily living: Katz Index, Barthel Index, IADL -Psychological state: Dementia Apathy Interview and Rating, EGD-15 (depression), Goldberg Anxiety and Depression Scale	**ADL:** **Wii group** ↑ Barthel (*p* = 0.025), ↓ Katz (*p* = 0.028), ↑ Lawton & Brody (*p* < 0.001) **Control** = stable or worsened **Cognition:** **Wii group** ↑ MCE (*p* < 0.001), ↓ GDS (*p* = 0.001) **Control** worsened **Psychological wellbeing:** ***Wii group* ↓** depression (EDG-15, *p* < 0.001), ↓ apathy (DAIR, *p* < 0.001), ↓ anxiety/depression (EADG, *p* < 0.001). ***Control* =** worsening depression and apathy **Physical well-being:** improvement in ADL functionality **Emotional well-being:** reduction in depression, anxiety and apathy **Mental well-being:** increase in cognition (memory, attention) **Engagement:** high satisfaction, good compliance, constant participation
10	Swinnen et al., 2021 [31] Belgium (Europe) Exergame (exercise programme; platform NR)	Randomised clinical trial To analyze the effects of an exergame exercise programme on physical, cognitive and emotional health and quality of life in elderly people with dementia living in long-term care facilities	35 participants divided into 2 groups: **EG** (23; 84.7 ± 5.6; 5 males and 18 females) **CG** (22; 85.3 ± 6.5; 5 males and 17 females)	The following variables were measured at T0 and at 8 weeks (T1): -Physical function: SPPB -Motor speed: SRTT -Cognitive function: MoCA -Behaviour: NPI -Depressive symptoms: CSDD -Quality of life: DQoL -Functional independence: Katz ADL	**Physical wellbeing:** significant improvements in gait and physical function **Cognitive wellbeing:** positive effects on cognitive function **Emotional wellbeing:** reduction in depressive symptoms **Quality of life:** moderate improvement, not statistically significant
11	Campo-Prieto et al., 2022 [32] Spain (Europe) Exergame (IVR exergame via HMD)	Randomized controlled clinical trial To assess the feasibility, usability and effects of IVR exergames on balance, mobility and risk of falls in nonagenarians	12 participants, all female, divided into 2 groups: **EG** (6; 91.67 ± 1.63 years) **CG** (6; 90.83 ± 2.64 years)	The following variables were measured at T0 and at 10 weeks (T1): -Functional mobility: TUG -Balance: Tinetti test -Participants’ experiences and usability of VR sessions: SSQ, SUS	**Usability:** SUS 78.3/100 (“good”); no symptoms of cybersickness (SSQ = 0). **Balance (Tinetti):** EG significant improvement Balance + 10.9% (*p* = 0.017), Gait + 9.2% (*p* = 0.047), Total + 10.2% (*p* = 0.014). CG worsened (balance − 15.2%; total − 9.3%) **Risk of falls:** EG went from moderate risk (22) to minimal risk (24.5 points) **Mobility (TUG):** EG stable (−0.45%), CG worsened (+14.8% total time)
12	Campo-Prieto et al., 2022 [33] Spain (Europe) Exergame (IVR exergame via HMD)	Randomised controlled clinical trial To examine the feasibility and effects of an IVR exergame on physical functions (balance, mobility, strength) and quality of life in institutionalized elderly people	24 participants divided into 2 groups: **EG** (13; 85.08 ± 8.48; 84.6% female) **CG** (11; 84.82 ± 8.10; 90.9% female)	The following variables were measured at T0 and at 10 weeks (T1): -Balance and gait: Tinetti test -Functional mobility and risk of falls: TUG -Quality of life: SF-12 -Cybersickness: SSQ -Usability: SUS -Post-game experiences: GHQ post-game module	**Feasibility**: no adverse events, no dropouts; good usability (SUS > 73/100); no cybersickness **Physical functions:** Significant ↑ in Tinetti (balance + 1.84; gait + 1.00; total + 2.84, *p* < 0.001) and Handgrip (+4.96 kg, *p* < 0.001). CG worsened in Tinetti and TUG **Quality of life:** both groups improved mental component; CG improved physical component more **Subjective well-being:** positive experiences (100% “good/excellent”); 81.8% would recommend the experience; reported benefits in agility, energy, motivation to exercise
13	Cuevas-Lara et al., 2022 [7] Spain (Europe) Gamification (in-hospital mobilization; simple vs. tech-based)	Controlled, open-label, non-randomized clinical trial To assess the effect of gamified interventions on functional capacity in hospitalized elderly people	70 participants (29 females and 41 males; 86.01 ± 4.27 years) divided into 3 groups: **Simple gamification group** (21) **Technology-based gamification group** (23) **CG** (26)	The following variables were assessed within 24 h of hospital admission (T0) and at discharge (T1): -Physical function: Short Physical Performance Battery (SPPB) and Barthel Index -Muscle strength: Handgrip strength -Global cognitive function: Mini-Mental State Examination (MMSE) -Mood: Yesavage Geriatric Depression Scale (15-item) -Quality of life: EuroQol-5 Dimension Questionnaire (EQ-5D-3L, with VAS)	**Functional capacity:** SPPB: +1.47 points (SGG) and +2.69 points (TGG) compared to control **Barthel Index:** +5.28 points (TGG) compared to control **Muscle strength:** significant increase in handgrip strength in the TGG (+2.03 kg) **Quality of life and mood:** not significant **Engagement:** high adherence, no adverse events, improved motivation with user-centred design **Physical well-being:** improved (SPPB, Barthel, handgrip) **Emotional and mental well-being:** no significant differences
14	Gunst et al., 2022 [34] Belgium (Europe) Exergame (nursing home programme; platform NR)	Randomised clinical trial Examine the effect of exergames on overall wellbeing, sleep quality, pain and cognitive function in nursing home residents. Also assess enjoyment, social interactions and feasibility	32 participants (22 females and 10 males; average age > 80 years) divided into an experimental group (15) and a control group (17)	The following variables were assessed at T0, 3 (T1) and 6 months (T2): -Executive function and attention: SC-WT -Visuospatial and cognitive assessment: CDT -Quality of life: OPQOL-35 -Physical functioning, pain, mental health, social role: RAND-36 -Cognitive decline: IQCODE-N)	**Mental well-being:** EG ↑ from 42/50 to 45 (vs. CG stable at 41) **Sleep:** EG ↑ 23 → 28/30, CG stable 24 → 25 **Pain:** EG ↑ 18 → 20/20, CG 17 → 16 **Cognition:** EG ↑ subjective 24 → 26/30, CG 23 → 27 **Engagement/Fun:** Average participation 88% Fun VAS ≈ 8.9/10 Borg intensity ≈ 11.6/20 (moderate effort) Enjoyment (4.4/5), would like to continue (4.4/5), socialization 2.8/5 (moderate) **Physical wellbeing:** perception of feeling fitter, slight reduction in pain. **Emotional wellbeing:** enjoyment, pleasure, feeling of novelty. **Social wellbeing:** increased socialization, but no new deep connections. **Quality of life:** improved in areas related to sleep, pain and perceived wellbeing. **Engagement:** very high, desire to continue.
15	Zheng et al., 2022 [35] China (Asia) Exergame (movement-based active games; platform NR)	Randomized clinical trial (pilot study) To assess the effect of exergames on cognition, quality of life and depression in elderly people with dementia	38 participants divided into 2 groups: **EG** (18; 15 females and 3 males; 81.74 ± 5.79) **CG** (20; 14 females and 6 males; 84.26 ± 5.48)	The following variables were measured at T0 and at 8 weeks (T1): -Cognitive functions: MMSE -Quality of life: QoL-AD -Depression: CSDD	**Cognition (MMSE):** EG ↑ from 14.06 ± 6.66 to 16.78 ± 7.18 CG unchanged (13.95 → 13.9) **Quality of life (QoL-AD):** EG ↑ from 28.17 ± 5.21 to 31.83 ± 3.99 (*p* = 0.023 intragroup) CG unchanged (28.00 → 27.25, *p* = 0.669) Significant difference between groups (*p* = 0.005, Cohen’s d = 0.96) **Depression (CSDD):** EG ↓ from 15.67 ± 5.88 to 7.61 ± 4.55 (*p* < 0.001 intragroup) CG worsened (15.75 → 17.6, *p* = 0.561) Difference between groups highly significant (*p* = 0.001, Cohen’s d = 1.229) **Emotional well-being:** significant reduction in depression, positive mood **Social well-being:** improved group socialization, mutual support and interaction with researchers during play **Quality of life:** significant perceived increase in QoL-AD **Engagement:** very high, 100% attendance, friendly competition, high satisfaction, no adverse events
16	Gallou-Guyot et al., 2023 [36] France (Europe) Exergame (dual-task programme)	Observational pilot study Assess the feasibility, safety and attractiveness of a dual-task exergame programme and analyze its potential effects on cognitive and motor functions, motivation to engage in physical activity, fear of falling and quality of life.	39 participants (29 females, 10 males; 84.6 ± 8.5 years)	The following variables were assessed at T0 and at 12 weeks (T1): -Executive functions: Stroop test -Functional mobility: TUG -Quality of life: EQ5D5L -Motivation: EMAPS	**Cognitive well-being**: significant improvements in working memory (N-Back, *p* < 0.001, d = 1.19) and cognitive inhibition in dual-task (Stroop errors ↓, *p* = 0.002). Emotional-affective well-being: activities perceived as engaging, positive and motivating Social participation: increased social interaction
17	Lee, 2023 [37] South Korea (Asia) Exergame (home-based programme; platform NR)	Randomised clinical trial To evaluate the effects of home-based exergame programmes on physical function, fall effectiveness, depression and health-related quality of life in community-dwelling older adults	57 participants divided into 2 groups: **EG** (28; 17 males and 11 females; 80.39 ± 2.57) **CG** (29; 14 males and 15 females; 79.10 ± 3.90)	The following variables were measured at T0 and at 8 weeks (T1): -Physical function: OLST, BBS, FRT, TUG, FTSTS, MFES -Depressive symptoms: GDS -Quality of life: SF-36	**Physical function:** significant improvements in EG compared to CG in OLST (*p* < 0.001) **Fall efficacy:** MFES ↑ in EG (*p* = 0.006) **Depression:** GDS ↓ in EG (*p* < 0.001) **Quality of life (SF-36):** Physical function ↑ in EG (*p* < 0.001) Role limitations physical ↑ in EG (*p* = 0.003) General health ↑ in EG (*p* = 0.010) Vitality ↑ in EG (*p* = 0.002) **Physical well-being:** improved lower limb strength, balance, mobility. **Emotional well-being:** reduced depression, improved vitality/energy **Mental well-being:** no significant differences in SF-36 **Social well-being:** no change in social functioning **Engagement:** high → good compliance, no drop-outs due to lack of motivation; game perceived as enjoyable and accessible
18	Brookman et al., 2024 [38] Australia (Oceania) Exergame (Road Worlds Competition for Seniors)	Pilot study, mixed methods Assessing the impact of an international cycling competition (26 days) as a multimodal intervention to improve the physical, psychological and social well-being of elderly people in residential care homes	32 participants (17 males and 15 females and 2 males; 83.06 ± 7.72 years old)	The following variables were assessed after the gaming period (2 weeks): -Lower limb strength, balance and functional mobility: 5STS -Functional endurance: 2MWT -Depressive symptoms: GDS-SF -Depressive symptoms: GDS-SF -Depressive symptoms in people with dementia: CSDD -Anxiety: GAI -Self-efficacy: GES -Social participation: LSNS -Participants’ perceptions and experiences: semi-structured interviews	**Physical wellbeing:** significant improvement in strength and physical fitness, residents report increased strength, endurance, independent walking, improved circulation. **Emotional wellbeing:** significant increase in self-efficacy post-intervention (*p* = 0.022) and reduction in depression (*p* = 0.034); reported improvements in happiness, relaxation, motivation, positive self-perception. **Social wellbeing:** strengthened bonds with staff, volunteers and other residents, sense of community. **Engagement:** perceived as very high (competition, scenarios, socializing). **Camaraderie:** team identity, reduced isolation, mutual motivation.
19	Samson et al., 2025 [39] Canada (North America) Serious game (semi-immersive music game)	Cross-sectional study Evaluating user experience with a semi-immersive music game aimed at preventing age-related neurocognitive decline	60 participants (41 females and 19 males; 84.5 ± 5.5 years)	The tools used assessed: -Cognitive status: MMSE -Level of engagement (5-level scale = interactive, constructive, active, passive and disengaged behaviours)	**Engagement:** 38% active, 35% constructive, 10% interactive, 17% passive; no ‘disengaged’ **Perceived well-being**: positive comments (‘it did me good’, ‘it brings comfort, memories, conviviality’) **Socialization:** the game encouraged dialogue with the therapist and between patients, with a strong connective value. **Quality of life:** not formally measured, but reported by patients as improved (hope, pleasure, relaxation). No symptoms of cybersickness.

***Note****:* ↑ improvement/increase; ↓ reduction; EG = experimental group; CG = control group; BBS-14 = Berg Balance Scale—14 items; TUG = Timed Up and Go Test; 6MWT = Six-Minute Walk Test; GDS-15 = Geriatric Depression Scale—15 items; SF-8 = Short Form Health Survey—8 items; FES = Falls Efficacy Scale; SEE = Self-Efficacy for Exercise Scale; BBS = Berg Balance Scale; NPI = Neuropsychiatric Inventory, 12 core symptom areas; SSQ = Symptom Severity Questionnaire, 27 items; TMT = Trail Making Test—25 items; DS = Digital Span; WMS-III = video game; OPQoL-Brief = Older Person’s Quality of Life—11 items; MCE = Mini-Cognitive Examination; NPI = Neuropsychiatric Inventory—12 items; IADL = Instrumental Activities of Daily Living Scale—8 items; EGD-15 = Geriatric Depression Scale-15 items; SPPB = Short Physical Performance Battery; SRTT = Step Reaction Time Test; MoCA = Montreal Cognitive Assessment; Katz ADL = Activities of Daily Living; Tinetti test = POMA Performance-Oriented Mobility Assessment—32 items; SUS = System Usability Scale—10 items; SF-12 = Short Form Health Survey—12 items; GHQ = General Health Questionnaire—12 items; MMSE = Mini-Mental State Examination—11 items; CDT = Clock Drawing Test; RAND-36 = health-related quality of life (HRQoL)—36-item; QoL-AD = Quality of Life in Alzheimer’s Disease—13 items; CSDD = Cornell Scale for Depression in Dementia—19 *items; EQ5D5L = EuroQol Five Dimensions Five Levels—5 items; EMAPS = Motivation Scale Towards Health-Oriented Physical Activity—30 items; SF-36 = Form Health Survey—36 items; OLST = One-Leg Standing Test; FRT = Functional Reach Test; FTSTS = Five Times Sit-to-Stand test; MFES = Modified Falls Efficacy Scale—14 items; 5STS = Five Times Sit-to-Stand Test; 2MWT = Two-Minute Walk Test; GAI = Geriatric Anxiety Inventory—20 items; GES = General Self Efficacy Scale—10 items; LSNS = Lubben Social Network Scale; NR = not reported; HMD = head-mounted display; IVR = immersive virtual reality; Wii Fit/Wii Fit Plus = Nintendo Wii exergames using a Balance Board/motion controllers; FRED = exergame developed for frail older adults; 360° VR reminiscence = HMD-based 360° environments/videos for memory stimulation.*

### 3.5. First Subquestion: How Gamified and Digital Interventions Are Used Among Adults Aged ≥75 Years

Across the 19 included studies, digital interventions were most often delivered as structured programmes integrating physical exercise and/or cognitive stimulation, and were frequently framed as strategies to enhance motivation, adherence, and engagement [22,26,31,38]. Implementation typically occurred in care settings (e.g., residential facilities, long-term care/nursing homes, assisted living), commonly under supervision by a physiotherapist, instructor, or staff member [22,23,26,31,33,38]. Only a small number of studies implemented home-based interventions among community-dwelling older adults [37].

Exergames were the most frequently used technology and were primarily designed to combine physical activity with game-based elements, targeting balance, mobility, fall risk and strength, and, in some cases, incorporating cognitive or dual-task components [22,26,31,37]. Several studies used motion-sensing platforms (e.g., Wii Fit/Wii Fit Plus) within programmes of varying duration (approximately 3–12 weeks, with some longer follow-up periods), delivered either individually or in groups within institutional settings [22,26,28]. Other investigations employed exergames developed specifically for frail populations (e.g., FRED) or implemented tailored protocols for people living with dementia [25,31]. A subset of exergame interventions also used immersive virtual reality (IVR), including among very old participants (e.g., nonagenarians), and incorporated dedicated assessments of usability and tolerability [32,33].

Serious games were mainly applied to support cognitive stimulation and to evaluate user experience. One study examined adoption and longer-term use of a mobile cognitive-stimulation app with automated usage tracking, while another evaluated a semi-immersive music game with a focus on engagement and the lived experience of play [24,39].

Digital games encompassed diverse approaches. One study used 360° VR headset content to stimulate memory and communication in people with dementia, while monitoring potential symptoms such as visual fatigue/cybersickness. Another tested brief action video game sessions among oldest-old residents in a care facility, focusing on attentional/executive outcomes and acceptability [27,29].

### 3.6. Second Subquestion: Reported Outcomes in Adults Aged ≥75 Years

**Figure 4:**
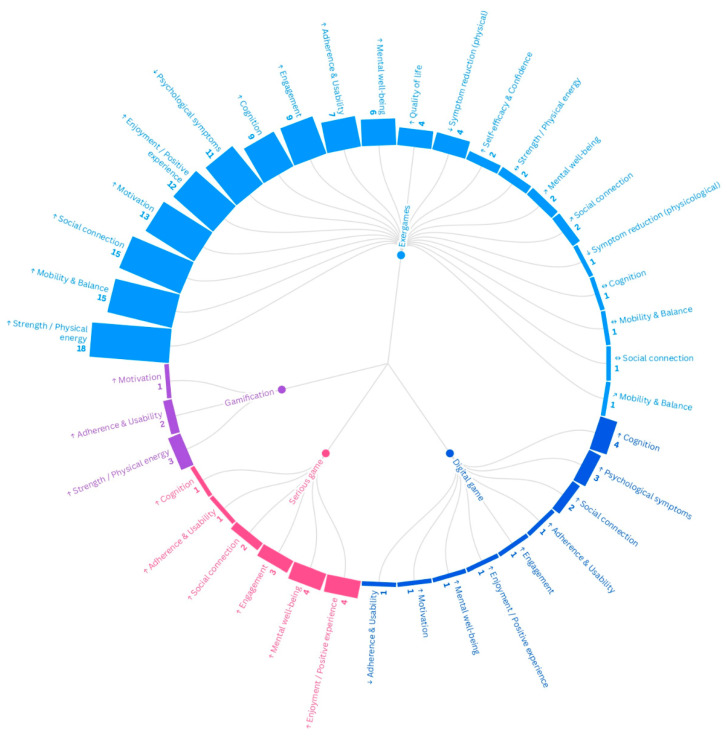
Radial chord diagram of reported outcomes by technology type in adults aged ≥75 years. **Note:** ↑ improvement/increase; ↓ reduction; *↔* no change; ↗ non-significant increase.

Across the 19 included studies, outcomes reported for adults aged ≥75 years clustered five main domains—physical, cognitive/mental, emotional/affective, social well-being, and quality of life—and frequently included implementation-related outcomes, particularly engagement/adherence and usability/feasibility. Figure 4 summarizes outcome frequency and direction by technology: bar height represents the number of articles reporting each outcome, while arrows indicate the direction of change described by study authors (↑, ↓, ↔, ↗), without implying effect size or certainty.

Physical outcomes were reported most frequently, particularly in exergame studies, with common measures of balance and mobility (e.g., BBS, TUG, Tinetti, SPPB) and indicators of strength. Several trials reported improvements in balance and/or functional mobility, although some measures remained stable or showed non-significant changes depending on the outcome measure and study design.

Cognitive/mental outcomes were reported less consistently and included attention/executive-function measures, performance on cognitive-stimulation tasks, and dementia-related cognitive/behavioural indicators. Studies using cognitive serious games or immersive/VR digital content often emphasized cognitive engagement, stimulation, and task performance, sometimes supported by usage metrics (e.g., session frequency, time spent playing, level progression).

Emotional/affective outcomes included depressive symptoms, enjoyment, motivation, and perceived psychological benefits. Some interventions reported reductions in depressive symptoms and increased enjoyment or sense of accomplishment, while other studies mainly described positive experiences without clear changes in symptom scales.

Social well-being outcomes included social interaction, bonding with peers or staff, and communication-related benefits. These outcomes were particularly evident in group-based institutional programmes and in dementia-focused VR reminiscence interventions, where spontaneous communication and shared memories were frequently described.

Quality of life (QoL) was assessed less consistently and was often treated as a secondary outcome. Where measured (e.g., SF-8/SF-12/EUROHIS), findings were mixed, including improvements in mental QoL components in some studies and modest or non-significant changes overall.

Regarding implementation outcomes, engagement and adherence were frequently reported, ranging from high acceptability and willingness to continue, to more moderate attendance levels in some institutional programmes. Usability outcomes were especially prominent in IVR/VR studies, which commonly reported favourable usability (e.g., SUS in the “good” range) and limited cybersickness/adverse events under supervised conditions.

### 3.7. Third Subquestion: To What Extent Do Studies Explicitly Stratify Their Samples or Results by Age (≥75 Years)?

During full-text assessment, 36 records were excluded because outcomes for participants aged ≥75 years were not reported separately, could not be extracted, or the age composition was described in insufficient detail to attribute findings to the ≥75 subgroup. The most common reporting limitations were the absence of age-stratified analyses, reporting only mean/median age without subgroup breakdown, and pooling outcomes across wide age ranges, which prevented extraction of ≥75-specific results.

A descriptive comparison between excluded and included evidence suggested that studies conducted in mixed older-adult samples (often broadly defined as ≥65 years) frequently evaluated a wide range of game-based and digital interventions. These included technology-supported exercise and rehabilitation (e.g., exergame therapy for hand function; digitally enabled mobility/rehabilitation programmes; exergaming combined with yoga in heart failure), as well as immersive or semi-immersive modalities (e.g., VR-based cognitive stimulation; fully immersive VR exergames with dual-task components in Parkinson’s disease). Mixed-sample studies also addressed socially oriented or engagement-focused technologies (e.g., assistive robots for socialization; multi-user serious games) and game-centred mobile applications targeting combined physical–cognitive performance. Across these excluded studies, outcomes—based on titles and screening information—most commonly emphasized physical function and rehabilitation endpoints (e.g., mobility, exercise capacity, functional performance), cognitive training/performance, and psychological or mental health outcomes (e.g., mood or depressive symptoms), alongside frequent attention to feasibility, usability/acceptance, and engagement.

By contrast, the included evidence more consistently supported interpretation for adults aged ≥75 years, because samples were predominantly oldest-old or provided extractable data for the ≥75 subgroup. Interventions in the included studies were often delivered in supervised care contexts and typically involved exergames (including IVR-based exergames) or gamified approaches. Reported outcomes most frequently captured balance, mobility, fall risk, and functional capacity, with additional reporting on enjoyment/motivation and social interaction. In IVR studies, usability and tolerability indicators (e.g., SUS/SSQ) were also commonly reported. Overall, this comparison suggests that research in broader older-adult samples spans a wide range of interventions and outcomes, whereas evidence that can be directly attributed to adults aged ≥75 years is more often concentrated in supervised exergame-based programmes and focuses on a narrower—yet clinically meaningful—set of physical-function and acceptability outcomes.

## 4. Discussion

This scoping review mapped 19 studies with data attributable to adults aged ≥75 years and indicates that the evidence base largely centred on structured, supervised programmes. Exergames emerged as the most commonly used technology, and physical-function outcomes were the endpoints most frequently assessed.

This pattern partly aligns with the expectation that game-based digital interventions are used to support well-being in very old age; however, it also suggests that “well-being” is often operationalized primarily through mobility, balance, fall risk, and functional capacity, whereas quality of life and social outcomes are less consistently prioritized and measured.

A further expectation was that digital/game-based interventions would be implemented across diverse contexts (community, home, and healthcare). In contrast, the included evidence was largely institutional and supervised, implying that feasibility and safety considerations (e.g., frailty, sensory deficits, fear of falling, cognitive impairment) may strongly influence where and how these interventions are delivered in the oldest-old.

The predominance of exergames and the frequent emphasis on balance and mobility is consistent with prior syntheses in broader “older adults” populations, which report improvements in mobility and balance and suggest that engagement and interactivity may help address common barriers to traditional exercise [16]. Nevertheless, much of the wider exergame literature aggregates participants across broad age ranges (often ≥65 years), meaning that evidence specific to adults aged ≥75—and especially those in the late 80s and 90s—remains less direct, even when the overall direction of findings aligns with the broader field.

For cognitive/mental and emotional outcomes, fewer studies were identified and findings were more heterogeneous. This generally accords with external evidence suggesting that serious games can improve certain cognitive outcomes and may reduce depressive symptoms, depending on the population and intervention characteristics [15]. At the same time, within ≥75 samples, cognitive and emotional effects appear to be captured through a mix of performance metrics, symptom scales, and acceptability/user-experience indicators rather than through consistently standardized, comparable endpoints.

The VR/IVR studies included—often prioritizing usability and tolerability while monitoring cybersickness—mirror a broader trend in the literature, whereby VR-based reminiscence and related immersive approaches are increasingly examined in older adults and dementia care. Reported benefits have included improvements in mood and engagement and selected cognitive outcomes, alongside close attention to tolerability [40]. Within the ≥75-focused evidence mapped here, usability findings (e.g., favourable SUS scores and low cybersickness under supervised conditions) therefore align with the direction of this evolving field, while also underscoring that effectiveness outcomes are not assessed uniformly across studies.

An important contribution of the ≥75 focus is the identification of a structural limitation in the literature: 36 full-text records were excluded because results for adults aged ≥75 were not stratified, could not be extracted, or age composition was insufficiently detailed to attribute outcomes to the oldest-old subgroup. Across excluded studies, non-extractability most often reflected a lack of subgroup analyses, age reported only as a mean/median without distribution, or outcomes pooled despite wide age ranges.

A descriptive comparison based on titles and screening information suggests that mixed-sample studies (often broadly defined as ≥65 years) span a wide and innovative range of digital/game-based approaches and outcomes—for example, technology-enabled rehabilitation and mobility programmes; exergaming combined with adjunct activities (e.g., yoga in clinical populations); immersive VR applications for neurological conditions; assistive robotics aimed at socialization; and game-centred mobile applications targeting combined physical–cognitive performance. By contrast, evidence directly attributable to adults aged ≥75 years appears more concentrated in supervised exergame programmes and tends to report a narrower—though clinically meaningful—set of outcomes (balance, mobility, fall risk, functional capacity), with variable inclusion of quality-of-life and social well-being measures. This gap has important interpretive implications: without routine age-disaggregated reporting, the broader “older adult” evidence base may obscure age-related differences in feasibility, engagement, safety, and responsiveness—issues that are especially consequential in the oldest-old, where frailty, multimorbidity, and cognitive impairment are more common.

From a global equity perspective, the evidence mapped in our review remains largely concentrated in high-income settings, whereas research on digital interventions for older adults in LMICs is still limited. A recent scoping review focusing on digital interventions for common mental health problems among older adults in LMICs identified only a small number of eligible studies, underscoring persistent gaps in availability, implementation, and reporting in these contexts. In addition, it is important to consider several equity and access barriers, such as digital literacy, sensory impairments, costs, and staffing resources, which often impact the feasibility of these interventions more than their effectiveness in the ≥75 population. These factors must be addressed to ensure the scalability and accessibility of digital interventions for the oldest-old, particularly in resource-constrained settings [41]. These constraints may be even more salient for game-based and VR approaches, which can require dedicated hardware, stable connectivity, and higher levels of digital literacy. Future research should therefore test scalable and context-adapted game-based solutions in LMIC settings and routinely report implementation and equity-relevant outcomes (e.g., reach, accessibility, feasibility, acceptability, and affordability), alongside health and well-being endpoints.

These findings are particularly relevant in light of global policy and research priorities on ageing. The UN Decade of Healthy Ageing (2021–2030) emphasizes optimizing functional ability and supporting participation and well-being through coordinated, scalable action [42].

Within this agenda, digital game-based interventions may represent a pragmatic route to promote activity, engagement, and person-centred care—particularly in settings with constrained staffing resources and among older adults who face barriers to conventional exercise or stimulation activities.

At the same time, the mapped evidence indicates that delivery for adults aged ≥75 years still relies heavily on supervised contexts. While this may reflect the need to ensure safety and adherence, it also highlights the need for further research on home- and community-based models, especially given increasing interest in usability and real-world implementation of home exergaming among older adults [6].

From a global equity perspective, the evidence mapped in our review remains largely concentrated in high-income settings, whereas research on digital interventions for older adults in LMICs is still limited. A recent scoping review focusing on digital interventions for common mental health problems among older adults in LMICs identified only a small number of eligible studies, underscoring persistent gaps in availability, implementation, and reporting in these contexts [43]. These constraints may be even more salient for game-based and VR approaches, which can require dedicated hardware, stable connectivity, and higher levels of digital literacy. Future research should therefore test scalable and context-adapted game-based solutions in LMIC settings and routinely report implementation and equity-relevant outcomes (e.g., reach, accessibility, feasibility, acceptability, and affordability), alongside health and well-being endpoints.

### Limitations of the Review

Although a formal risk-of-bias appraisal is not required for scoping reviews, the mapped evidence should be interpreted cautiously. Many included studies had small samples and short follow-up periods, often using pilot or feasibility designs. In addition, heterogeneity in populations, intervention protocols, delivery formats, and outcome measures limits comparability and the strength of inferences about effects.

Several issues should be considered when interpreting the findings of this scoping review. Consistent with scoping review methodology, no formal critical appraisal of methodological quality or risk of bias was undertaken; accordingly, the purpose was to map the available evidence rather than to draw conclusions about effectiveness or certainty of effects. Although the review focused on adults aged ≥75 years, age-specific synthesis was limited by the scarcity of age-disaggregated reporting in the primary literature, as reflected by the exclusion of 36 full-text records due to non-extractable ≥75 data. Heterogeneity in intervention types (e.g., exergames, serious games, digital games, gamification), delivery formats (individual vs. group; supervised vs. home-based), and outcome measures also reduced comparability across studies and likely contributed to variability in reported findings. In addition, the search was limited to peer-reviewed publications in English, which may have resulted in the omission of relevant programme evaluations, implementation reports, or studies published in other languages. Finally, the included evidence was geographically concentrated, which may limit transferability to health and social care systems with different resource levels, digital infrastructures, or cultural patterns of technology use in later life.

## 5. Implications for Research and Future Directions

Taken together, the mapped evidence suggests a clear research agenda for game-based and VR-supported interventions in adults aged ≥75 years. First, the field is constrained by limited age-disaggregated reporting: a substantial number of full texts could not be used because outcomes for the ≥75 subgroup were not extractable, which limits interpretability and transferability to the oldest-old. Routine reporting of age-stratified results (e.g., ≥75 vs. <75), alongside key descriptors such as frailty status, comorbidity burden, cognitive impairment, and digital literacy, is therefore essential to enable meaningful comparisons and equity-informed implementation.

Second, outcome measurement remains uneven. Physical-function endpoints dominate, while person-centred outcomes (quality of life, perceived well-being, social participation) are assessed less consistently despite their relevance to healthy ageing priorities. Future studies should adopt more multidimensional and standardized outcome sets, combining clinical endpoints with person-centred measures and implementation indicators (adherence, acceptability, usability, safety).

Third, the predominance of supervised, institutional delivery highlights an implementation gap: comparative and pragmatic studies are needed to test which delivery models are safe, acceptable, and sustainable for different profiles of very old adults. This includes evaluating supervised versus scalable home- and community-based formats (including hybrid approaches), and specifying the training and support required for participants and/or caregivers.

Finally, for immersive technologies, future research should more systematically report VR-specific implementation and tolerability parameters (e.g., exposure time/dose, usability, cybersickness, adverse events), and clarify which game elements drive engagement and outcomes in cognitively or physically vulnerable subgroups. Addressing these priorities will strengthen the evidence base and support the development of targeted, feasible, and person-centred game-based interventions for adults aged ≥75 years.

## 6. Conclusions

This scoping review indicates that evidence on game-based digital interventions among adults aged ≥75 years is currently concentrated in supervised settings and is dominated by exergame-based programmes, with outcomes most frequently reported in the physical domain. By contrast, quality of life and social well-being are assessed less consistently, despite their central importance to healthy ageing. A major challenge in this field is the limited availability of age-disaggregated results: many studies conducted in mixed older-adult samples do not report outcomes in ways that allow extraction for the ≥75 subgroup, constraining age-specific inference. Importantly, this scoping review maps evidence attributable to adults aged ≥75 years and does not support robust comparisons across an age gradient (e.g., ≥75 vs. <75).

To strengthen the international evidence base and make findings actionable for the oldest-old, future research should implement routine age-stratified reporting, adopt more consistent and multidimensional outcome measurement (including quality of life and social well-being), and standardize reporting of implementation, safety, and tolerability—particularly for immersive technologies and clinically vulnerable subgroups. Pragmatic, context-sensitive studies that compare supervised institutional delivery with scalable home- and community-based models, and that tailor technologies to frailty, dementia, and multimorbidity, are needed to clarify which interventions work best, for whom, and under what conditions.

## Data Availability

No new data were created or analyzed in this study.

## Supplementary Materials

The following supporting information can be downloaded at: https://www.mdpi.com/article/10.3390/healthcare14040470/s1, Supplementary Table S1: PCC Framework; Supplementary Table S2: Search strategies on database; Supplementary Table S3: Exclusion of Full-Text studies with motivation.

## References

[1] United Nations World Population Ageing, 2019: Highlights 2019. https://digitallibrary.un.org/record/3846855/files/WorldPopulationAgeing2019-Highlights.pdf.

[2] United Nations World Population Prospects 2022: Summary of Results|Population Division 2022. https://www.un.org/development/desa/pd/content/World-Population-Prospects-2022.

[3] World Health Organization World Report on Ageing and Health 2015. https://www.who.int/publications/i/item/9789241565042.

[4] Chu C.H., Quan A.M.L., Souter A., Krisnagopal A., Biss R.K. (2022). Effects of Exergaming on Physical and Cognitive Outcomes of Older Adults Living in Long-Term Care Homes: A Systematic Review. Gerontology.

[5] Vasodi E., Saatchian V., Dehghan Ghahfarokhi A. (2023). Virtual reality-based exercise interventions on quality of life, some balance factors and depression in older adults: A systematic review and meta-analysis of randomized controlled trials. Geriatr. Nur..

[6] de Medeiros C.S.P., Farias L.B.A., Santana M.C.d.L., Pacheco T.B.F., Dantas R.R., Cavalcanti F.A.d.C. (2024). A systematic review of exergame usability as home-based balance training tool for older adults usability of exergames as home-based balance training. PLoS ONE.

[7] Gao Y., Liu N. (2025). Effects of digital technology-based serious games interventions for older adults with mild cognitive impairment: A meta-analysis of randomised controlled trials. Age Ageing.

[8] Stavropoulou I., Sakellari E., Barbouni A., Notara V. (2025). Community-Based Virtual Reality Interventions in Older Adults with Dementia and/or Cognitive Impairment: A Systematic Review. Exp. Aging Res..

[9] Holt-Lunstad J., Smith T.B., Baker M., Harris T., Stephenson D. (2015). Loneliness and Social Isolation as Risk Factors for Mortality: A Meta-Analytic Review. Perspect. Psychol. Sci..

[10] Marmot M., Friel S., Bell R., Houweling T.A., Taylor S. (2008). Closing the gap in a generation: Health equity through action on the social determinants of health. Lancet.

[11] Martinho D., Carneiro J., Corchado J.M., Marreiros G. (2020). A systematic review of gamification techniques applied to elderly care. Artif. Intell. Rev..

[12] Yoong S.Q., Wu V.X., Jiang Y. (2024). Experiences of older adults participating in dance exergames: A systematic review and meta-synthesis. Int. J. Nurs. Stud..

[13] Mądra-Gackowska K., Szewczyk-Golec K., Gackowski M., Hołyńska-Iwan I., Parzych D., Czuczejko J., Graczyk M., Husejko J., Jabłoński T., Kędziora-Kornatowska K. (2025). Selected Biochemical, Hematological, and Immunological Blood Parameters for the Identification of Malnutrition in Polish Senile Inpatients: A Cross-Sectional Study. J. Clin. Med..

[14] Mądra-Gackowska K., Szewczyk-Golec K., Gackowski M., Woźniak A., Kędziora-Kornatowska K. (2023). Evaluation of Selected Parameters of Oxidative Stress and Adipokine Levels in Hospitalized Older Patients with Diverse Nutritional Status. Antioxidants.

[15] Saragih I.D., Everard G., Lee B.-O. (2022). A systematic review and meta-analysis of randomized controlled trials on the effect of serious games on people with dementia. Ageing Res. Rev..

[16] Pacheco T.B.F., de Medeiros C.S.P., de Oliveira V.H.B., Vieira E.R., de Cavalcanti F.a.C. (2020). Effectiveness of exergames for improving mobility and balance in older adults: A systematic review and meta-analysis. Syst. Rev..

[17] Saragih I.D., Suarilah I., Saragih I.S., Lin Y.-K., Lin C.-J. (2024). Efficacy of serious games for chronic pain management in older adults: A systematic review and meta-analysis. J. Clin. Nurs..

[18] Peters M.D.J., Marnie C., Tricco A.C., Pollock D., Munn Z., Alexander L., McInerney P., Godfrey C.M., Khalil H. (2020). Updated methodological guidance for the conduct of scoping reviews. JBI Evid. Synth..

[19] Tricco A.C., Lillie E., Zarin W., O’Brien K.K., Colquhoun H., Levac D., Moher D., Peters M.D.J., Horsley T., Weeks L. (2018). PRISMA Extension for Scoping Reviews (PRISMA-ScR): Checklist and Explanation. Ann. Intern. Med..

[20] Lommi M., Ivziku D., Guarente L., Vanzi V. Gamification, Exergames, and Digital Games in Older Adults Aged 75 and Over: Age-Dependent Effects on Well-being, Quality of Life, and Social Engagement—A Scoping Review. OSF 2026. https://osf.io/2hymj/overview.

[21] Ouzzani M., Hammady H., Fedorowicz Z., Elmagarmid A. (2016). Rayyan—A web and mobile app for systematic reviews. Syst. Rev..

[22] Chao Y.-Y., Scherer Y.K., Montgomery C.A., Wu Y.-W., Lucke K.T. (2015). Physical and Psychosocial Effects of Wii Fit Exergames Use in Assisted Living Residents: A Pilot Study. Clin. Nurs. Res..

[23] Chao Y.-Y., Lucke K.T., Scherer Y.K., Montgomery C.A. (2016). Understanding the Wii Exergames Use: Voices from Assisted Living Residents. Rehabil. Nurs..

[24] Yasini M., Marchand G. (2016). Adoption and Use of a Mobile Health Application in Older Adults for Cognitive Stimulation. Transforming Healthcare with the Internet of Things.

[25] Mugueta-Aguinaga I., Garcia-Zapirain B. (2017). FRED: Exergame to Prevent Dependence and Functional Deterioration Associated with Ageing. A Pilot Three-Week Randomized Controlled Clinical Trial. Int. J. Environ. Res. Public Health.

[26] Taylor L., Kerse N., Klenk J., Borotkanics R., Maddison R. (2018). Exergames to Improve the Mobility of Long-Term Care Residents: A Cluster Randomized Controlled Trial. Games Health J..

[27] Coelho T., Marques C., Moreira D., Soares M., Portugal P., Marques A., Ferreira A.R., Martins S., Fernandes L. (2020). Promoting Reminiscences with Virtual Reality Headsets: A Pilot Study with People with Dementia. Int. J. Environ. Res. Public Health.

[28] García-Bravo S., García-Bravo C., Molina-Rueda F., Cuesta-Gómez A. (2020). Training with Wii Balance Board for Dynamic Balance in Older Adults. Top. Geriatr. Rehabil..

[29] McCord A., Cocks B., Barreiros A.R., Bizo L.A. (2020). Short video game play improves executive function in the oldest old living in residential care. Comput. Hum. Behav..

[30] Jahouh M., González-Bernal J.J., González-Santos J., Fernández-Lázaro D., Soto-Cámara R., Mielgo-Ayuso J. (2021). Impact of an Intervention with Wii Video Games on the Autonomy of Activities of Daily Living and Psychological–Cognitive Components in the Institutionalized Elderly. Int. J. Environ. Res. Public Health.

[31] Swinnen N., Vandenbulcke M., de Bruin E.D., Akkerman R., Stubbs B., Firth J., Vancampfort D. (2021). The efficacy of exergaming in people with major neurocognitive disorder residing in long-term care facilities: A pilot randomized controlled trial. Alzheimers Res. Ther..

[32] Campo-Prieto P., Cancela-Carral J.M., Rodríguez-Fuentes G. (2022). Feasibility and Effects of an Immersive Virtual Reality Exergame Program on Physical Functions in Institutionalized Older Adults: A Randomized Clinical Trial. Sensors.

[33] Campo-Prieto P., Cancela-Carral J.M., Alsina-Rey B., Rodríguez-Fuentes G. (2022). Immersive Virtual Reality as a Novel Physical Therapy Approach for Nonagenarians: Usability and Effects on Balance Outcomes of a Game-Based Exercise Program. J. Clin. Med..

[34] Gunst M., De Meyere I., Willems H., Schoenmakers B. (2021). Effect of exergaming on wellbeing of residents in a nursing home: A single blinded intervention study. Aging Clin. Exp. Res..

[35] Zheng J., Yu P., Chen X. (2022). An Evaluation of the Effects of Active Game Play on Cognition, Quality of Life and Depression for Older People with Dementia. Clin. Gerontol..

[36] Gallou-Guyot M., Mandigout S., Marie R., Robin L., Daviet J.-C., Perrochon A. (2023). Feasibility and potential cognitive impact of a cognitive-motor dual-task training program using a custom exergame in older adults: A pilot study. Front. Aging Neurosci..

[37] Lee K. (2023). Home-Based Exergame Program to Improve Physical Function, Fall Efficacy, Depression and Quality of Life in Community-Dwelling Older Adults: A Randomized Controlled Trial. Healthcare.

[38] Brookman R., Hulm Z., Hearn L., Siette J., Mathew N., Deodhar S., Cass A., Smith J., Kenny B., Liu K.P.Y. (2024). Evaluation of an exercise program incorporating an international cycling competition: A multimodal intervention model for physical, psychological, and social wellbeing in residential aged care. BMC Geriatr..

[39] Samson L., Carcreff L., Noublanche F., Noublanche S., Vermersch-Leiber H., Annweiler C. (2025). User Experience of a Semi-Immersive Musical Serious Game to Stimulate Cognitive Functions in Hospitalized Older Patients: Questionnaire Study. JMIR Serious Games.

[40] Ng W.H.D., Ang W.H.D., Fukahori H., Goh Y.S., Lim W.S., Siah C.J.R., Seah B., Liaw S.Y. (2024). Virtual reality-based reminiscence therapy for older adults to improve psychological well-being and cognition: A systematic review. J. Clin. Nurs..

[41] Yang S., Xu W., Chen K. (2025). Digital literacy and its effects on older adults’ health: Exploring mechanisms and outcomes. Front. Public Health.

[42] World Health Organization WHO’s Work on the UN Decade of Healthy Ageing (2021–2030). https://www.who.int/initiatives/decade-of-healthy-ageing?utm_source=chatgpt.com.

[43] Saha I., Sundström C., Kandasamy A., Kraepelien M., Dahiya N., Saha A., Jayaram-Lindström N., Chakrabarti A., Benegal V. (2025). Digital interventions for common mental health problems among older adults in low- and middle-income countries: A scoping review. BMJ Glob. Health.

