# Supplementary material for: Gamification, Exergames, and Digital Games in Older Adults Aged 75 and Over: Evidence on Well-Being, Quality of Life, and Social Engagement—A Scoping Review
Source: Healthcare (Basel). 2026 Feb 12;14(4):470. doi: 10.3390/healthcare14040470 (PMC12940992; doi:10.3390/healthcare14040470)
Supplement: Supplementary file 1 [file healthcare-14-00470-s001.zip › healthcare-4095747-supplementary.pdf]

Supplementary Table S1: PCC Framework

Scope	Details	Keywords e MeSH
Population	Adults aged ≥65 years, including subgroup reporting or extractable data for ≥75 years.	Aged[MeSH] OR Elderly[MeSH] OR "older adult*" OR "aging population" OR senior*
Concept	Interventions involving gamification, serious games, exergames, or digital games<br><br>AND<br><br>Outcomes related to quality of life, socialization, and well-being.	Gamification OR Exergaming[MeSH] OR Exergame* OR serious game* OR digital game*<br><br>AND<br><br>Quality of Life[MeSH] OR Life Quality OR QOL OR Socialization OR social interaction OR well-being OR social engagement
Context	Community, home, residential, or healthcare settings, without geographical restrictions.	

Supplementary Table S2: Search strategies on database

Database	Query search	Records
**PubMed**	(aged OR elderly OR "older adult*" OR "aging population" OR senior*) AND (gamification OR exergam* OR "serious game*" OR "digital game*") AND (“quality of life” OR “life quality” OR QOL OR socialization OR well-being OR "social interaction")	**101**
**CINAHL**	XB (aged or elderly or senior or older people or older adult or aging population) AND XB (gamification or exergames or exergaming or serious games or digital games) AND XB (quality of life or well being or well-being or socialization or social interaction)	**162**
**PsycInfo**	AB (aged or elderly or senior or older people or older adult or aging population) AND AB (gamification or exergames or exergaming or serious games or digital games) AND AB (quality of life or well being or well-being or socialization or social interaction)	**44**
**Scopus**	TITLE-ABS-KEY (aged OR elderly OR "older adults" OR "aging population" OR seniors) AND TITLE-ABS-KEY (gamification OR exergaming OR exergames OR "serious game*" OR "digital games") AND TITLE-ABS-KEY (quality AND of AND life OR life AND quality OR socialization OR QOL OR well-being OR "social interaction")	**554**
**WOS**	TS=(aged or elderly or senior or older people or older adult or aging population)) AND TS=(gamification or exergames or exergaming or serious games or digital games) AND TS=(quality of life or well being or well-being or socialization or social interaction)	**385**

Filters: Publication date (last 10 years), Language (English), Species (Humans), Target population (aged).

Supplementary Table S3: Exclusion of Full-Text studies with motivation

N.	Reference	Reason for exclusion
1	Akdemir, S., Tarakci, D., Budak, M., & Hajebrahimi, F. (2023). The effect of leap motion controller based exergame therapy on hand function, cognitive function and quality of life in older adults: A randomised trial. Journal of Gerontology and Geriatrics, 71(3), 152–165. https://doi.org/10.36150/2499-6564-N606	The results were not stratified by age (≥75 vs. <75 years)
2	Hassett, L., van den Berg, M., Lindley, R. I., Crotty, M., McCluskey, A., van der Ploeg, H. P., Smith, S. T., Schurr, K., Howard, K., Hackett, M., Killington, M., Bongers, B., Togher, L., Treacy, D., Dorsch, S., Wong, S., Scrivener, K., Chagpar, S., Weber, H., … Sherrington, C. (2020). Digitally enabled aged care and neurological rehabilitation to enhance outcomes with Activity and MObility UsiNg Technology (AMOUNT) in Australia: A randomised controlled trial. PLOS Medicine, 17(2), e1003029. https://doi.org/10.1371/journal.pmed.1003029	The results were not stratified by age (≥75 vs. <75 years)
3	Zheng, H., Li, J., Salmon, C. T., & Theng, Y.-L. (2020). *The effects of exergames on emotional well-being of older adults. Computers in Human Behavior, 110*, 106383. https://doi.org/10.1016/j.chb.2020.106383	The results were not stratified by age (≥75 vs. <75 years)
4	Klompstra, L., Hägglund, E., Jaarsma, T., Kato, N. P., & Strömberg, A. (2025). Effects of exergaming and yoga on exercise capacity and physical and mental health in heart failure patients: A randomized sub-study. European Journal of Cardiovascular Nursing, 24(3), 389–398. https://doi.org/10.1093/eurjcn/zvae155	The results were not stratified by age (≥75 vs. <75 years)
5	Pereira, F., Bermúdez i Badia, S., Ornelas, R., & Cameirão, M. S. (2019). Impact of game mode in multi-user serious games for upper limb rehabilitation: A within-person randomized trial on engagement and social involvement. Journal of NeuroEngineering and Rehabilitation, 16(1), 1–15. https://doi.org/10.1186/s12984-019-0536-2	The results were not stratified by age (≥75 vs. <75 years)
6	Gamito, P., Oliveira, J., Alves, C., Santos, N., Coelho, C., & Brito, R. (2020). Virtual reality-based cognitive stimulation to improve cognitive functioning in community elderly: A controlled study. Cyberpsychology, Behavior, and Social Networking, 23(12), 1–7. https://doi.org/10.1089/cyber.2019.0271	The results were not stratified by age (≥75 vs. <75 years)

7	D'Onofrio, G., Fiorini, L., Hoshino, H., Matsumori, A., Okabe, Y., Tsukamoto, M., Limosani, R., Vitanza, A., Greco, F., Greco, A., Giuliani, F., Cavallo, F., & Sancarlo, D. (2019). *Assistive robots for socialization in elderly people: Results pertaining to the needs of the users. Aging Clinical and Experimental Research, 31*(9), 1313–1329. https://doi.org/10.1007/s40520-018-1073-z	The results were not stratified by age (≥75 vs. <75 years)
8	Lee, M.-A., Ferraro, K. F., & Kim, G. (2021). Digital technology use and depressive symptoms among older adults in Korea: Beneficial for those who have fewer social interactions? Aging & Mental Health, 25(10), 1839–1847. https://doi.org/10.1080/13607863.2020.1839863	The results were not stratified by age (≥75 vs. <75 years)
9	Shake, M. C., Crandall, K. J., Mathews, R. P., Falls, D. G., & Dispennette, A. K. (2018). Efficacy of Bingocize®: A game-centered mobile application to improve physical and cognitive performance in older adults. Games for Health Journal, 7(4), 253–261. https://doi.org/10.1089/g4h.2017.0139	The results were not stratified by age (≥75 vs. <75 years)
10	Lauzé, M., Martel, D. D., Agnoux, A., Sirois, M.-J., Émond, M., Daoust, R., & Aubertin-Leheudre, M. (2018). Feasibility, acceptability and effects of a home-based exercise program using a gerontechnology on physical capacities after a minor injury in community-living older adults: A pilot study. Journal of Nutrition, Health & Aging, 22(1), 16–25. https://doi.org/10.1007/s12603-017-0890-1	The results were not stratified by age (≥75 vs. <75 years)
11	Yun, S. J., Hyun, S. E., Oh, B.-M., & Seo, H. G. (2023). Fully immersive virtual reality exergames with dual-task components for patients with Parkinson's disease: A feasibility study. Journal of NeuroEngineering and Rehabilitation, 20(92). https://doi.org/10.1186/s12984-023-01215-7	The results were not stratified by age (≥75 vs. <75 years)
12	Zeleníková, R., Hosáková, J., Kozáková, R., Bobčíková, K., & Bužgová, R. (2025). *The effect of reminiscence therapy on the assessment of depression, anxiety and self-esteem in community-dwelling older adults: An intervention study. International Journal of Older People Nursing, 20*, e70004. https://doi.org/10.1111/opn.70004	The results were not stratified by age (≥75 vs. <75 years)
13	Brill, E., Holfelder, A., Falkner, M., Krebs, C., Brem, A.-K., & Klöppel, S. (2024). *Behavioural and neuronal substrates of serious game-based computerised cognitive training in cognitive decline: Randomised controlled trial. BJPsych Open, 10*(e200), 1–9. https://doi.org/10.1192/bjo.2024.797	The results were not stratified by age (≥75 vs. <75 years)

14	Cicek, A., Ozdincler, A. R., & Tarakci, E. (2020). Interactive video game-based approaches improve mobility and mood in older adults: A nonrandomized, controlled trial. Journal of Bodywork and Movement Therapies, 24(3), 252–259. https://doi.org/10.1016/j.jbmt.2020.01.005	The results were not stratified by age (≥75 vs. <75 years)
15	Schell, R., Hausknecht, S., Zhang, F., & Kaufman, D. (2016). Social benefits of playing Wii Bowling for older adults. Games and Culture, 11(1–2), 81–103. https://doi.org/10.1177/1555412015607313	The results were not stratified by age (≥75 vs. <75 years)
16	Chen, C.-K., Tsai, T.-H., Lin, Y.-C., Lin, C.-C., Hsu, S.-C., Chung, C.-Y., … Hsu, Y.-W. (2018). Acceptance of different design exergames in elders. PLoS ONE, 13(7), e0200185. https://doi.org/10.1371/journal.pone.0200185	The results were not stratified by age (≥75 vs. <75 years)
17	Mugueta-Aguinaga, I., & Garcia-Zapirain, B. (2019). Frailty level monitoring and analysis after a pilot six-week randomized controlled clinical trial using the FRED exergame including biofeedback supervision in an elderly day care centre. International Journal of Environmental Research and Public Health, 16(19), 3359. https://doi.org/10.3390/ijerph16193359	The results were not stratified by age (≥75 vs. <75 years)
18	Çiçek, A., Ozdincler, A. R., & Tarakci, E. (2020). Interactive video game-based approaches improve mobility and mood in older adults: A nonrandomized, controlled trial. Journal of Bodywork & Movement Therapies, 24(2), 252–259. https://doi.org/10.1016/j.jbmt.2020.01.005	The results were not stratified by age (≥75 vs. <75 years)
19	Carballeira, E., Censi, K. C., Maseda, A., López-López, R., Lorenzo-López, L., & Millán-Calenti, J. C. (2021). Low-volume cycling training improves body composition and functionality in older people with multimorbidity: A randomized controlled trial. International Journal of Environmental Research and Public Health, 18(16), 8690. https://doi.org/10.3390/ijerph18168690	The results were not stratified by age (≥75 vs. <75 years)
20	Wang, C.-M., & Huang, C.-S. (2023). Using digital technology to design a simple interactive system for nostalgic gaming to promote the health of slightly disabled elderly people. International Journal of Environmental Research and Public Health, 20(1), 128. https://doi.org/10.3390/ijerph20010128	The results were not stratified by age (≥75 vs. <75 years)
21	Drazich, B. F., Crane, B. M., Taylor, J. L., Szanton, S. L.,	The results were

	Moore, K. D., Eldreth, D., Ahmad, O., Krakauer, J. W., Resnick, B., & Carlson, M. C. (2023). Older adults' subjective well-being experiencing the exergame "I Am Dolphin." Games for Health Journal, 12(2), 131–139. https://doi.org/10.1089/g4h.2022.0092	not stratified by age (≥75 vs. <75 years)
22	Manser, P., Poikonen, H., & de Bruin, E. D. (2023). Feasibility, usability, and acceptance of "Brain-IT"—A newly developed exergame-based training concept for the secondary prevention of mild neurocognitive disorder: A pilot randomized controlled trial. Frontiers in Aging Neuroscience, 15, 1163388. https://doi.org/10.3389/fnagi.2023.1163388	The results were not stratified by age (≥75 vs. <75 years)
23	Park, J., Hung, L., Randhawa, P., Surage, J., Sullivan, M., Levine, H., & Ortega, M. (2023). 'Now I can bend and meet people virtually in my home': The experience of a remotely supervised online chair yoga intervention and visual socialisation among older adults with dementia. Health & Social Care in the Community, 31(6), e6291–e6302. https://doi.org/10.1111/hsc.14264	The results were not stratified by age (≥75 vs. <75 years)
24	Rossetto, F., Isernia, S., Realdon, O., Borgnis, F., Blasi, V., Pagliari, C., Cabinio, M., Alberoni, M., Mantovani, F., Clerici, M., & Baglio, F. (2023). A digital health home intervention for people within the Alzheimer's disease continuum: Results from the Ability-TelerehABILITation pilot randomized controlled trial. BMC Health Services Research, 23, 608. https://doi.org/10.1186/s12913-023-09598-0	The results were not stratified by age (≥75 vs. <75 years)
25	Esnard, C., Haza, M., & Grangeiro, R. (2024). Older people in the world of esport: A qualitative study. Frontiers in Psychology, 15, 1460966. https://doi.org/10.3389/fpsyg.2024.1460966	The results were not stratified by age (≥75 vs. <75 years)
26	Tuan, S.-H., Chang, L.-H., Sun, S.-F., Li, C.-H., Chen, G.-B., & Tsai, Y.-J. (2024). Assessing the clinical effectiveness of an exergame-based exercise training program using Ring Fit Adventure to prevent and postpone frailty and sarcopenia among older adults in rural long-term care facilities: Randomized controlled trial. Journal of Medical Internet Research, 26, e54546. https://doi.org/10.2196/54546	The results were not stratified by age (≥75 vs. <75 years)
27	Schell, R., Hausknecht, S., Zhang, F., & Kaufman, D. (2016). Social benefits of playing Wii Bowling for older adults. Games and Culture, 11(1–2), 81–103. https://doi.org/10.1177/1555412015607313	The results were not stratified by age (≥75 vs. <75 years)

28	Chen, C.-K., Tsai, T.-H., Lin, Y.-C., Lin, C.-C., Hsu, S.-C., Chung, C.-Y., … Hsu, Y.-W. (2018). Acceptance of different design exergames in elders. PLoS ONE, 13(7), e0200185. https://doi.org/10.1371/journal.pone.0200185	The results were not stratified by age (≥75 vs. <75 years)
29	Mugueta-Aguinaga, I., & Garcia-Zapirain, B. (2019). Frailty level monitoring and analysis after a pilot six-week randomized controlled clinical trial using the FRED exergame including biofeedback supervision in an elderly day care centre. International Journal of Environmental Research and Public Health, 16(19), 3359. https://doi.org/10.3390/ijerph16193359	The results were not stratified by age (≥75 vs. <75 years)
30	Çiçek, A., Ozdincler, A. R., & Tarakci, E. (2020). Interactive video game-based approaches improve mobility and mood in older adults: A nonrandomized, controlled trial. Journal of Bodywork & Movement Therapies, 24(2), 252–259. https://doi.org/10.1016/j.jbmt.2020.01.005	The results were not stratified by age (≥75 vs. <75 years)
31	Carballeira, E., Censi, K. C., Maseda, A., López-López, R., Lorenzo-López, L., & Millán-Calenti, J. C. (2021). Low-volume cycling training improves body composition and functionality in older people with multimorbidity: A randomized controlled trial. International Journal of Environmental Research and Public Health, 18(16), 8690. https://doi.org/10.3390/ijerph18168690	The results were not stratified by age (≥75 vs. <75 years)
32	Wang, C.-M., & Huang, C.-S. (2023). Using digital technology to design a simple interactive system for nostalgic gaming to promote the health of slightly disabled elderly people. International Journal of Environmental Research and Public Health, 20(1), 128. https://doi.org/10.3390/ijerph20010128	The results were not stratified by age (≥75 vs. <75 years)
33	Drazich, B. F., Crane, B. M., Taylor, J. L., Szanton, S. L., Moore, K. D., Eldreth, D., Ahmad, O., Krakauer, J. W., Resnick, B., & Carlson, M. C. (2023). Older adults' subjective well-being experiencing the exergame "I Am Dolphin." Games for Health Journal, 12(2), 131–139. https://doi.org/10.1089/g4h.2022.0092	The results were not stratified by age (≥75 vs. <75 years)
34	Manser, P., Poikonen, H., & de Bruin, E. D. (2023). Feasibility, usability, and acceptance of "Brain-IT"—A newly developed exergame-based training concept for the secondary prevention of mild neurocognitive disorder: A pilot randomized controlled trial. Frontiers in Aging Neuroscience, 15, 1163388. https://doi.org/10.3389/fnagi.2023.1163388	The results were not stratified by age (≥75 vs. <75 years)

35	Park, J., Hung, L., Randhawa, P., Surage, J., Sullivan, M., Levine, H., & Ortega, M. (2023). 'Now I can bend and meet people virtually in my home': The experience of a remotely supervised online chair yoga intervention and visual socialisation among older adults with dementia. Health & Social Care in the Community, 31(6), e6291–e6302. https://doi.org/10.1111/hsc.14264	The results were not stratified by age (≥75 vs. <75 years)
36	Rossetto, F., Isernia, S., Realdon, O., Borgnis, F., Blasi, V., Pagliari, C., Cabinio, M., Alberoni, M., Mantovani, F., Clerici, M., & Baglio, F. (2023). A digital health home intervention for people within the Alzheimer's disease continuum: Results from the Ability-TelerehABILITation pilot randomized controlled trial. BMC Health Services Research, 23, 608. https://doi.org/10.1186/s12913-023-09598-0	The results were not stratified by age (≥75 vs. <75 years)
